# Identification and Validation of a Urinary Biomarker Panel to Accurately Diagnose and Predict Response to Therapy in Lupus Nephritis

**DOI:** 10.3389/fimmu.2022.889931

**Published:** 2022-05-30

**Authors:** Laura Whittall-Garcia, Kirubel Goliad, Michael Kim, Dennisse Bonilla, Dafna Gladman, Murray Urowitz, Paul R. Fortin, Eshetu G. Atenafu, Zahi Touma, Joan Wither

**Affiliations:** ^1^ Division of Rheumatology, Department of Medicine, Toronto Western Hospital, University of Toronto, Toronto, ON, Canada; ^2^ Schroeder Arthritis Institute, Krembil Research Institute, University Health Network, Toronto, ON, Canada; ^3^ University of Toronto Lupus Clinic, Centre for Prognosis Studies in the Rheumatic Diseases, Toronto Western Hospital, University Health Network, Toronto, ON, Canada; ^4^ Department of Immunology, Faculty of Medicine, University of Toronto, Toronto, ON, Canada; ^5^ Division of Rheumatology, Department of Medicine, Centre de recherche du CHU de Québec–Université Laval, Quebec City, QC, Canada; ^6^ Department of Biostatistics, University Health Network, Toronto, ON, Canada

**Keywords:** predictors of response, urinary biomarker, biomarkers, lupus nephritis, systemic lupus erythematosus

## Abstract

**Background:**

We have previously shown that 15 urinary biomarkers (of 129 tested by Luminex), discriminate between active Lupus Nephritis (ALN) and non-LN patients. The aim of this study was to evaluate the ability of these 15 previously-identified urinary biomarkers to predict treatment responses to conventional therapy, and for the most predictive of these biomarkers to validate their utility to identify ALN patients in an independent prospectively-acquired lupus cohort.

**Methods:**

Our study had a 3-stage approach. In stage 1, we used Luminex to examine whether our previously identified urinary biomarkers at the time of the renal flare ( ± 3 months) or 12 ± 3 months after treatment of biopsy-proven ALN could predict treatment responses. In stage 2, a larger prospectively-acquired cross-sectional cohort was used to further validate the utility of the most predictive urinary biomarkers (identified in stage 1) to detect ALN patients. In this 2^nd^ stage, cut-offs with the best operating characteristics to detect ALN patients were produced for each biomarker and different combinations and/or numbers of elevated biomarkers needed to accurately identify ALN patients were analyzed. In stage 3, we aimed to further corroborate the sensitivity of the cut-offs created in stage 2 to detect ALN patients in a biopsy-proven ALN cohort who had a urine sample collection within 3 months of their biopsy.

**Results:**

Twenty-one patients were included in stage 1. Twelve (57.1%), 4 (19.1%), and 5 (23.8%) patients had a complete (CR), partial (PR) and no (NR) remission at 24 ± 3 months, respectively. The percentage decrease following 12 ± 3 months of treatment for Adiponectin, MCP-1, sVCAM-1, PF4, IL-15 and vWF was significantly higher in patients with CR in comparison to those with PR/NR. In stage 2, a total of 247 SLE patients were included, of which 24 (9.7%) had ALN, 79 (31.9%) had LN in remission (RLN) and 144 (58.3%) were non-LN (NLN) patients. Based on the combinations of biomarkers with the best operating characteristics we propose “rule out” and “rule in” ALN criteria. In stage 3, 53 biopsy-proven ALN patients were included, 35 with proliferative LN and 18 with non-proliferative ALN, demonstrating that our “rule in ALN” criteria operate better in detecting active proliferative than non-proliferative classes.

**Conclusions:**

Our results provide further evidence to support the role of Adiponectin, MCP-1, sVCAM-1 and PF4 in the detection of proliferative ALN cases. We further show the clinical utility of measuring multiple rather than a single biomarker and we propose novel “rule in” and “rule out” criteria for the detection of proliferative ALN with excellent operating characteristics.

## Introduction

Lupus nephritis (LN) occurs in up to 65% of patients with Systemic Lupus Erythematosus (SLE), and is most prevalent in younger patients, many of whom are of African, Asian, and Hispanic ancestry (1–3). LN is one of the most common causes of death as well as an important predictor of subsequent mortality in SLE (3–8). It is also associated with a significant morbidity, since up to 20% of patients will progress to end stage renal disease (3, 9), which has a particularly high socioeconomic impact (10, 11).

The gold standard for determining the presence and type of kidney involvement is the kidney biopsy (KB) (12). However, serial biopsies to assess renal activity following treatment are impractical due to their invasive nature and risk of complications. There is also a subset of patients with contraindications that preclude a KB at the time of LN flare. Consequently, the diagnosis of LN and the monitoring of response to treatment has been based on urinary findings of proteinuria, hematuria, pyuria, or casts, and alterations of renal function, such as increased serum creatinine.

The utility of proteinuria as a biomarker has drawbacks. LN-associated proteinuria frequently persists for years after renal injury, especially in patients with nephrotic range proteinuria, normalizing in less than 50% of patients within two years (13). In addition, proteinuria may reflect chronic histologic lesions rather than active inflammation within the kidney, as demonstrated by Malvar et al. who showed that 62% of LN patients who had complete histologic remission on a repeat KB following initiation of therapy were still ‘clinically active’, as defined by persistent proteinuria (14). Being able to correctly differentiate between residual activity and damage in LN is crucial when treating patients, highlighting the need for new biomarkers in the clinical setting.

Various urinary cytokines, chemokines, pro-inflammatory factors, growth factors and adhesion molecules, have been assessed as potential urinary biomarkers for LN (15–24). Unfortunately, none of them have been able to successfully transition into clinical practice, with the lack of clear cut-offs and algorithms that accurately detect active LN (ALN) being part of the challenge. We have previously shown that 42 urine biomarkers (of 129 tested by Luminex) discriminate between ALN and non-LN patients (NLN). Of these, Clusterin, Cystatin C, NGAL, PF4, vWF, sVCAM-1, GM-CSF, GRO, IL-15, IL-6, MCP-1, Adiponectin, PAI-1, MMP-7 and TIMP-1 were the 15 biomarkers with the most promising results, based on their ability to discriminate between ALN and non-active LN (remission LN, RLN) and/or their correlations with histologic features in the KB (16).

The aim of this study was to evaluate the ability of these 15 previously identified urinary biomarkers to predict treatment responses following initiation of conventional therapy, and for the most predictive of these biomarkers to validate their utility to accurately detect ALN in an independent prospectively acquired lupus cohort.

## Material and Methods

### Study Design and Patients

Patients with Systemic Lupus Erythematosus (SLE) from the University of Toronto Lupus cohort and the LuNNET cohort (16, 25) were included in the study. All patients met the revised 1997 ACR classification criteria for SLE (26) or had three criteria and a supportive biopsy (skin or kidney).

The study had 3 stages. In stage 1, we used Luminex to examine whether the 15 urinary biomarkers identified in our previous study could predict treatment response. For this stage, the cohort was composed of SLE patients from the LuNNET cohort, recruited from April 2006 to December 2011, all of whom had biopsy proven ALN. The KB was performed ± 3 months from the baseline urine sample collection, with all patients being followed longitudinally for a minimum of 2 years at the University of Toronto Lupus Clinic. Follow-up urine samples were collected every 3-6 months up to 24 ± 3 months.

The response to treatment was established after 24 months of follow-up, using the following criteria: 1) Complete response (CR): reduction in a 24 hour protein excretion to <500 mg/day with normal serum creatinine or serum creatinine within 15% of previous baseline; 2) Partial response (PR): > 50% reduction in proteinuria and to non-nephrotic levels, with serum creatinine within 25% of previous baseline; and 3) No response (NR): patients who did not achieve CR or PR (2, 27). Samples from 24 healthy controls were also assayed to enable determination of normal biomarker values.

In stage 2 of the study, the most predictive urinary biomarkers for response to treatment that were identified in stage 1 (Adiponectin, MCP-1, sVCAM, PF4, IL-15 and vWF) were assayed using ELISA, to further validate their ability to accurately detect ALN patients. For this second part of the study, a larger cross-sectional cohort was acquired. SLE patients from the University of Toronto Lupus cohort (enrolled within the last 5 years, to assure no overlap with the LuNNET cohort) were consecutively recruited from July 2016 to March 2019, when attending their scheduled clinic appointment. For this cohort, ALN was defined clinically as a LN flare that occurred within the last 12 months from the urine collection, with a 24 hour urinary protein excretion of ≥500mg/day, which was interpreted by the physician in charge as being secondary to active renal inflammation prompting a change in immunosuppressive therapy. Non-ALN patients were divided into two groups: 1) Patients with RLN, defined as the presence of a history of LN but no clinical signs of renal activity at the time of sample collection, with a 24 hour urinary protein excretion of <500mg/day or the presence of chronic proteinuria which was interpreted by the physician in charge as being secondary to damage and not requiring a change in immunosuppressive therapy. Chronic proteinuria was defined as stable proteinuria present for at least 1 year, in the presence of chronic kidney disease (CKD, defined as a eGFR<60ml/min/m^2^) and/or other comorbidities known to cause of proteinuria, such as diabetes mellitus and hypertension; and 2) NLN patients, with no history of LN and no clinical signs of ALN (urinary protein excretion <500mg/day) at the time of the urine sampling, but who could have extra-renal SLE activity.

In Stage 3, we aimed to validate the sensitivity of our urinary biomarker cut-offs, established in the cross-sectional cohort, to identify ALN patients, and determine if they operated similarly for proliferative and non-proliferative ALN classes. For this stage, urinary Adiponectin, MCP-1, sVCAM and PF4 were measured using ELISA. All patients had biopsy proven ALN ± 3 months from the urine sample collection.

### Urinary Biomarker Assays

All urine samples were spun to remove cellular debris and frozen at –80° C. To avoid repeated freeze/thaws, samples were thawed once on ice, sub-aliquoted, re-frozen at -80°C, and then individual aliquots thawed immediately prior to use. For the first stage of the study, the urinary concentrations of 15 analytes (Clusterin, Cystatin C, NGAL, PF4, vWF, sVCAM-1, GM-CSF, GRO, IL-15, IL-6, MCP-1, Adiponectin, PAI-1, MMP-7 and TIMP-1) were measured by coupled bead assay (Luminex using MILLIPLEX^®^ Map Kits (EMC Millipore Corporation) through Eve Technologies Inc.). Further information regarding the sensitivity and dynamic range of the assays can be found on the company website. For the majority of assays, the urine samples were run undiluted except for Clusterin and Cystatin C, which were diluted 1/50 and TIMP-1, which was diluted 1/5. All analytes were measured in duplicate, with a single sample on each of two separate plates and averaged. Urinary biomarker levels were considered abnormal if they were > 2 SD above the mean of the 24 healthy controls.

For the second and third stage of the study, sVCAM-1 (Cat# DY809), MCP-1 (Cat# DY279), Adiponectin (Cat# DY1065), PF-4 (Cat# DY795), vWF (Cat# DY2764-05) and IL-15 (Cat# DY247) were measured by ELISA, using Duoset and Ancillary Reagent Kits (Cat# DY008) obtained from R&D Systems, and processed following the manufacturer’s protocols. Optimal dilutions for each cytokine ELISA were determined in preliminary experiments and were 1/16 for Adiponectin, 1/128 for sVCAM-1, 1/8 for MCP-1, and 1/4 for PF-4, vWF, and IL-15. For the majority of samples IL-15 and vWF concentrations were below the limit of detection and therefore were not pursued further. All samples were run in duplicate, averaged, and their cytokine concentration computed from a ln–ln plot of the cytokine standard curve, with adjustment for the dilution factor. Any samples with raw absorbance values that were under the lower limit of the standard curve using the optimal dilution, were re-run at lower dilutions and those that were below the standard curve at a 1/4 dilution were given the lowest standard curve value for ensuing calculations.

### Statistical Analysis

Descriptive statistics were generated for patients’ baseline characteristics for the two cohorts, with baseline categorical variables being presented as counts and percentages. Continuous biomarker variables are presented as median and IQR or mean and standard deviation, as appropriate.

In the first stage of the study, logistic regression models were used to determine if the baseline urinary biomarker levels, or the absolute or percentage decrease, after 12 months of therapy predicted CR to treatment at 24 months. For this analysis non-CR (PR and NR) were pooled together. A scatter plot of each of the patient measurements at different time points, as well as a smooth line were plotted to visualize the trend of the curve depending on the response (CR *vs* PR/NR).

In the second stage of the study, the Kruskal-Wallis test was used to assess the differences in biomarker measures between groups. Logistic regression models were used to assess the impact of each of the potential continuous predictors to discriminate between ALN and non-ALN. A binary partitioning method was used to obtain the optimum cut-off for each biomarker that discriminated between ALN and non-ALN (RLN and NLN). Receiver Operating Characteristic curves were generated for each individual biomarker.

Sensitivity, specificity, positive predictive value (PPV), negative predictive value (NPV), positive likelihood ratio (+LR) and negative likelihood ratios (-LR) with 95% confidence intervals were calculated to determine the accuracy of detecting active LN when: 1 or more, 2 or more or 3 or more biomarkers were elevated. Sensitivity, specificity, PPV and NPV above 80% were considered good and above 90% excellent. Likelihood ratios above 10 for +LR and below 0.1 for –LR were considered to provide strong evidence to rule in or rule out diagnoses (28).

In the third stage of the study, the sensitivity with 95% confidence intervals was calculated for the presence of 2 or more, or 3 or more elevated biomarkers.

All p-values were 2-sided and for the statistical analyses, a p < 0.05 was considered to indicate a statistically significant result. Statistical analysis was performed using version 9.4 of the SAS system for Windows, Copyright ^©^ 2002-2012 SAS Institute, Inc., Cary, NC.

## Results

### Only a Subset of Urinary Biomarkers Demonstrate Change Over Time That Associates With Treatment Response

In the first stage of the study, 21 biopsy-proven ALN patients were included, 19 (90.5%) of whom had proliferative LN (see **Table 1**). The mean age at baseline was 32.15 years and 85.7% of patients were female. The predominant ethnicity was Caucasian (47.6%), followed by Asian (23.8%) and Afro-Caribbean (14.3%). The mean SLE disease duration was 3.69 years and the average time since the start of the LN flare to the urine sample collection was 1.19 ± 1.12 months. Twelve (57.1%), 4 (19.1%), and 5 (23.8%) patients had a complete (CR), partial (PR) and no remission (NR), respectively after 24 months of conventional therapy.

**Table 1:** Baseline demographic and clinical characteristics of the patient cohorts†.

	Stage 1	Stage 2 cross-sectional cohort N = 247		
	ALN (N = 21)	ALN (N = 24)	RLN (N = 79)	NLN (N = 144)
**Ethnicity, n (%)**				
Caucasian	10 (47.6)	8 (33.3)	41 (51.8)	82 (56.9)
Afro-Caribbean	3 (14.3)	9 (37.5)	14 (17.7)	33 (23.1)
Asian	5 (23.8)	4 (16.6)	11 (13.9)	12 (8.4)
Other	3 (14.3)	3 (12.5)	13 (16.5)	17 (11.9)
**Female, n (%)**	18 (85.7)	20 (83.3)	64 (81.0)	132 (91.6)
**Age (years), Median (IQR)**	28.9 (23.5-44.0)	28.6 (24.6-36.1)	41 (28.9-52.4)	38 (29.6-52.7)
**Duration SLE (years), Median (IQR)**	2.9 (0.1-7.5)	7.7 (3.5-10.2)	9.27 (4.2-17.5)	7.1 (2.8-13.5)
Time from LN flare (months)*, Median (IQR)	1.0 (0-2.0)	5.5 (2.7-10)	48 (24-108)	NA
**Antiphospholipid syndrome, n (%) **	1 (4.8)	1 (4.2)	9 (11.4)	9 (6.3)
**Hypertension, n (%)**	5 (23.8)	6 (25.0)	22 (27.8)	19 (13.2)
**Diabetes Mellitus, n (%)**	0 (0)	0 (0)	6 (7.6)	3 (2.1)
**Clinical features, n (%)**				
Mucocutaneous	7 (33.3)	4 (16.7)	8 (10.1)	13 (9.0)
Musculoskeletal	8 (38.1)	0 (0)	2 (2.5)	7 (4.9)
Serositis	2 (9.5)	0 (0)	0 (0)	1 (0.7)
Hematologic	3 (14.3)	1 (4.2)	6 (7.6)	13 (9.0)
Central Nervous System	0 (0)	0 (0)	1 (1.3)	1 (0.7)
Vasculitis	1 (4.8)	0 (0)	1 (1.26)	0 (0)
Renal	21 (100)	24 (100)	0 (0)	0 (0)
Fever	2 (9.5)	0 (0)	1 (1.3)	0 (0)
**SLEDAI, total score, Median (IQR)**	18 (14-24)	10 (6-13)	3 (0-4)	2 (0-4)
**SLEDAI, renal, Median (IQR)**	12 (8-12)	4 (4-8)	–	–
**Anti-dsDNA Ab (IU/ml), Median (IQR)**	100 (19-101)	55 (24-254)	15 (1-55)	3 (1-15)
**Positive Antiphospholipid Abs, n (%)**	4 (19.0)	4 (16.7)	18 (22.8)	33 (22.9)
**C3, g/L, Median (IQR)**	0.62 (0.35-0.77)	0.86 (0.67-0.99)	0.99 (0.82-1.12)	1.04 (0.85-1.22)
**C4, g/L, Median (IQR)**	0.07 (0.05-0.14)	0.14 (0.13-0.20)	0.17 (0.13-0.23)	0.19 (0.14-0.25)
**Serum Albumin (g/L), Median (IQR)**	29 (22-32)	34 (31.5-37.5)	41 (38-43)	42 (39-44)
**Serum Creatinine (umol/L), Median (IQR)**	89 (71-134)	77.5 (66.5-100)	77 (57-81)	64.5 (56.5-74.5)
**eGFR<60 ml/min/m^2^, n (%)**	6 (28.6)	6 (25)	11 (13.9)	0 (0)
**eGFR<30 ml/min/m^2^, n (%)**	3 (14.3)	2 (8.3)	3 (3.8)	0 (0)
**eGFR<15 ml/min/m^2^ or RRT, n (%)**	0 (0)	0 (0)	0	0 (0)
**24-hour Protein excretion (g), Median (IQR)**	2.1 (1.5-3.8)	1.1 (0.7-2.7)	0 (0-0.4)	0 (0-0.4)
**Kidney biopsy Class, n (%)**	21 (100)	11 (45.8)^#^		
I	0 (0)	0 (0)		
II	0 (0)	0 (0)		
III	1 (4.8)	2 (0.08)		
IV	10 (47.6)	5 (0.2)		
V	2 (9.5)	1 (0.04)		
III+V	4 (19)	1(0.04)		
IV + V	4 (19)	2 (0.08)		
VI	0 (0)	0 (0)		
**Activity Index, Median (IQR)**	11 (6-13)	7 (2.3-9.8)		
**Chronicity Index, Median (IQR)**	3 (2-4)	3 (2.3-4.8)		
**Prednisone, n (%)**	21 (100)	22 (91.7)	47 (59.5)	71 (49.3)
**Prednisone dose (mg), Median (IQR)**	45 (40-50)	15 (10-20)	5 (5-10)	4 (5-7.5)
**Antimalarial, n (%)**	20 (95.2)	20 (83.3)	67 (84.8)	124 (86.1)
**Immunosuppressive, n (%)**	21 (100)	23 (95.8)	56 (70.8)	83 (57.6)
**Azathioprine, n (%)**	3 (14.3)	1 (4.2)	11 (13.9)	31 (21.5)
**Azathioprine dose (mg), Median (IQR)**	125 (100/150)	100 (100/100)	100 (100/150)	100(75/150)
**Mychophenolate, n (%)**	16 (76.2)	20 (83.3)	42 (53.2)	37 (25.7)
**Mychophenolate dose (g), Median (IQR)**	2 (2-3)	2 (2-3)	2 (1.5-2.5)	2 (2-3)
**Cyclophosphamide, n (%)**	2 (19.5)	0 (0)	0 (0)	0 (0)
**Methotrexate, n (%)**	–	1(4.2)	3 (3.8)	15 (10.4)
**Methotrexate dose (mg), Median (IQR)**	–	22.5 (20-25)	12.5 (10-15)	17.5 (15-20)

^†^Baseline clinical characteristics are at the time of the urine sample collection, *Time from LN flare to urine sample collection (months), ^#^Remaining 13 patients did not have a KB at the time of the urine sample collection, of whom 3 did not have a prior KB and 10 had a prior KB (9 class III or IV with or without class V and 1 pure class V).

eGFR, estimated Glomerular Filtration Rate, RRT, Renal Replacement Therapy.

Patients who achieved CR, PR and NR were treated similarly. The dose of prednisone used at baseline was similar for the 3 groups (45, 52 and 39 mg for CR, PR and NR, respectively, p=0.241). In the CR group 2 (16%) were treated with Azathioprine, 2 (16%) with Cyclophosphamide and 8 (66.6%) with Mycophenolate. All patients with PR and 4 (80%) of the patients with NR were treated with Mycophenolate. The remaining NR patient was treated with Azathioprine.

**Figure 1:**
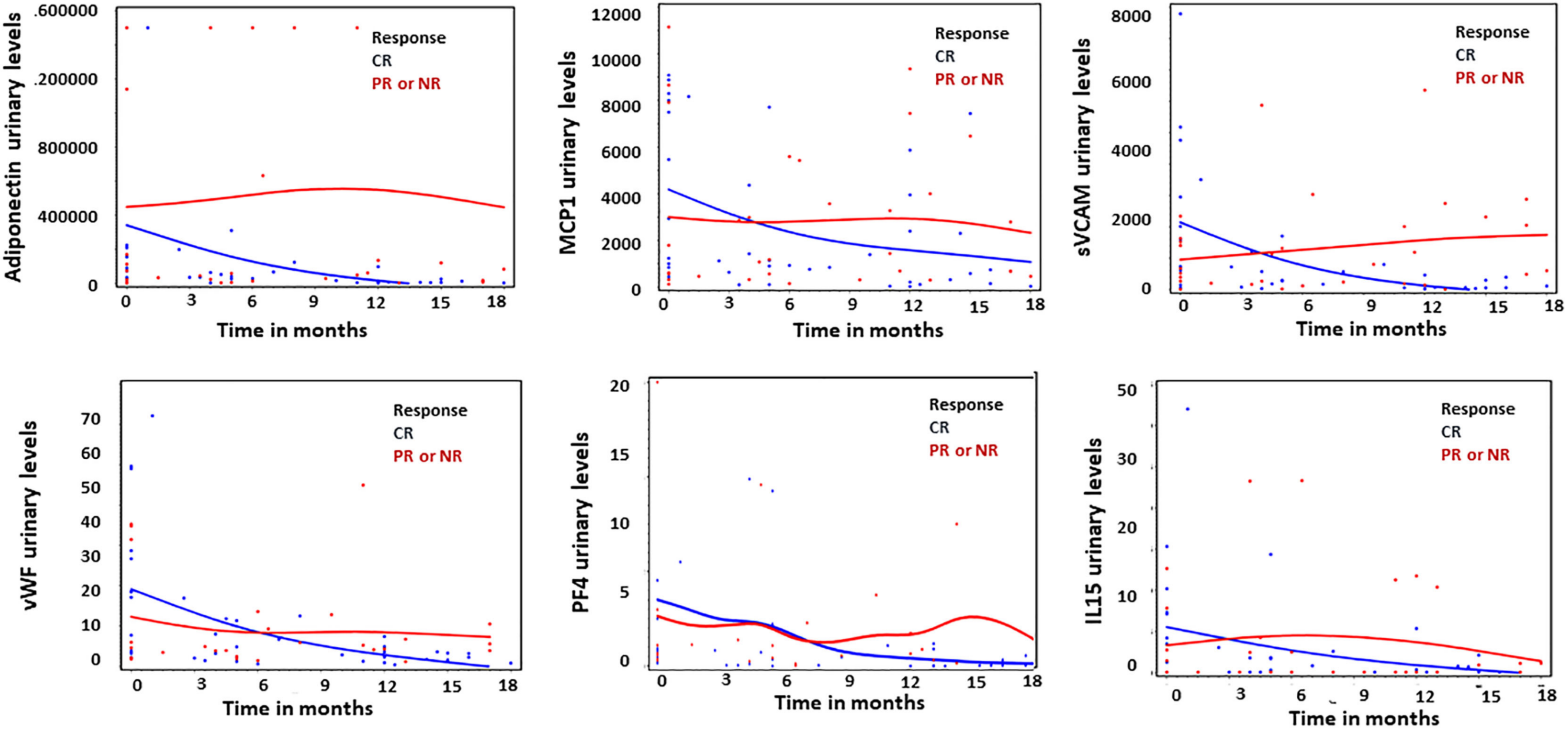
Thumbnail plots illustrating the difference in the amount of urinary biomarkers over time between complete responders (CR; n = 12, blue) and partial or non responders (PR or NR; n = 9, red). Units for all graphs are in pg/μmol, except PF4 expressed in ng/μmol.

The baseline levels of urinary biomarkers did not predict response to therapy at 24 months. However, the percentage decrease in Adiponectin, MCP-1, sVCAM-1, PF4, IL-15 and vWF at 12 ± 3 months predicted response to therapy at 24 months. (**Figure 1** and **Table 2**).

**Table 2:** Logistic regression analysis assessing baseline levels and percentage decrease at 12 ± 3 months as predictors of complete response at month 24 for the urinary biomarkers. N = 21.

	Baseline		% Decrease at month 12	
Biomarkers	OR (95% CI)	P value	OR (95% CI)	P value
Adiponectin	1.00 (1.00-1.00)	0.58	NA^1^	NA^1^
MCP-1	1.00 (1.00-1.00)	0.79	NA^2^	NA^2^
sVCAM-1	1.00 (1.00-1.00)	0.53	0.05 (0.006-0.44)	0.007
PF4	0.97 (0.84-1.13)	0.71	0.042 (0.004-0.49)	0.011
vWF	1.02 (0.96-1.08)	0.56	0.045 (0.004-0.54)	0.014
IL-15	1.09 (0.89-1.34)	0.41	0.143 (0.02-0.93)	0.042
Cystatin-C	0.99 (0.97-1.00)	0.21	1.00 (1.00-1.00)	0.94
PAI-1	0.99 (0.99-1.00)	0.23	0.98 (0.96-1.00)	0.13
GM-CSF	1.29 (0.83-1.98)	0.25	1.00 (1.00-1.00)	0.52
Lipocalin	0.99 (0.99-1.00)	0.52	1.00 (1.00-1.00)	0.87
GRO	0.99 (0.99-1.00)	0.69	0.99 (0.99-1.00)	0.36
MMP-7	1.00 (1.00-1.00)	0.72	0.99 (0.99-1.00)	0.86
IL-6	0.99 (0.97-1.02)	0.86	0.99 (0.99-1.00)	0.73
Clusterin	1.00 (1.00-1.00)	0.90	1.00 (0.99-1.00)	0.94
TIMP-1	0.99 (0.98-1.01)	0.92	0.99 (0.99-1.00)	0.14

NA^1^ – Not Applicable due to perfect specificity.

NA^2^ – Not Applicable due to perfect sensitivity.

### Validation of the Most Predictive Biomarkers in a Cross-Sectional SLE Cohort

A total of 247 SLE patients from the University of Toronto Lupus Clinic were included in stage 2, of whom 24 (9.7%) had ALN, 79 (31.9%) had RLN and 144 (58.3%) had NLN patients. All ALN patients were within 12 months of detection of the LN flare, with a mean time of 6 months between the initiation of the flare and the urine collection. Since our criteria for including ALN were clinical, only 11 (45.8%) had a KB at the time of their LN flare, of whom 10 (41.7%) had a proliferative class, either III or IV with or without class V, and 1 (0.04%) had pure membranous class V. The remaining 13 patients did not have a KB performed at the time of the flare. However, 10 had a prior KB (9 class III or IV with or without class V, and 1 pure class V). **Table 1** shows the baseline demographic and clinical characteristics of the cohort.

**Figure 2:**
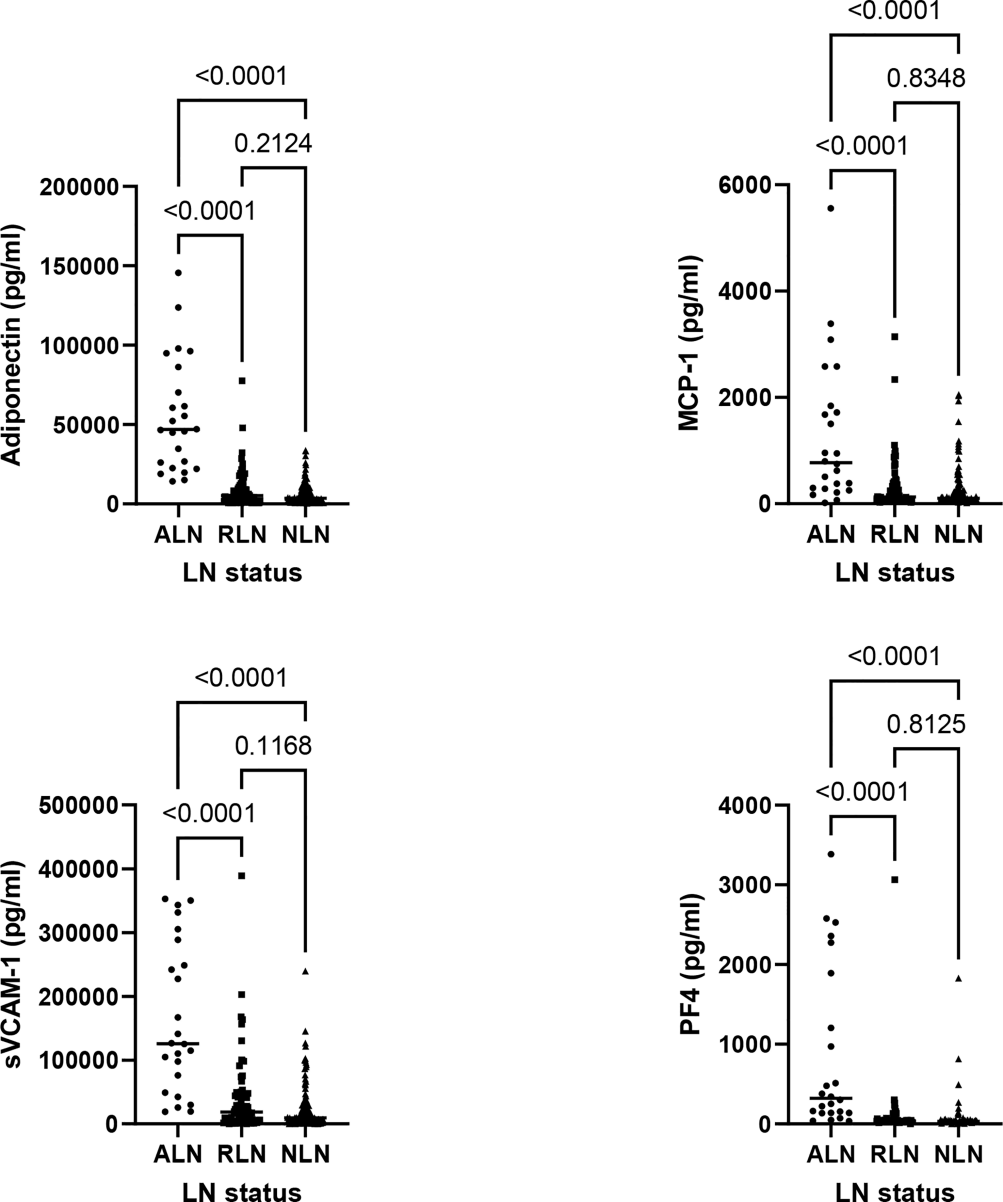
Comparison of biomarker levels between active LN (ALN; n = 24, circles), remission LN (RLN; n = 79, squares) and non LN (NLN; n=144, triangles). For all graphs each symbol represents the determination from a single individual, with the median value for each group indicated by a horizontal line. The Kruskal-Wallis test was used to assess the differences in biomarker levels between ALN, RLN and NLN patients.

Based on the findings from stage 1, 6 urinary biomarkers were measured by ELISA in the cross-sectional cohort, including Adiponectin, MCP-1, sVCAM-1, PF4, vWF and IL-15. vWF and IL-15 were not consistently detectable by ELISA and were not furthered studied. Patients with ALN had higher levels of all 4 remaining analytes, including Adiponectin, MCP-1, sVCAM-1 and PF4, in comparison to patients with RLN and NLN, as shown in **Figure 2** and **Supplementary Table 1**. Cut-offs with the best operating characteristics to detect ALN patients were produced for each biomarker (Adiponectin 18000 pg/ml, MCP-1 1341 pg/ml, sVCAM-1 46000 pg/ml and PF4 134 pg/ml), see **Supplementary Figure 1** for Receiver Operating Characteristic curves. Adiponectin was the most sensitive, with a high NPV (99%) and good -LR (0.09). However, 2 patients that were classified as ALN did not meet the Adiponectin cut-off. These patients were in the 4^th^ and 10^th^ month of the onset of their LN flare. In addition, Adiponectin alone had a low PPV (52%). In contrast, MCP-1 and PF4 had high specificities, but low PPV´s. Thus, no single biomarker appeared to be sufficient to accurately detect ALN patients (**Table 3**).

**Table 3:** Operating characteristics for individual biomarkers.

Biomarkers	Sensitivity (95% CI)	Specificity (95% CI)	PPV (95% CI)	NPV (95% CI)	+LR (95% CI)	-LR (95% CI)
**Adiponectin** (18000 pg/ml)	91.7 (73.0-99.0)	90.9 (86.3-94.3)	52.4 (36.4-68.0)	99.0 (96.5-99.9	10 (6.50-16)	0.09 (0.02-0.35)
**MCP-1** (1341 pg/ml)	37.5 (18.8-59.4)	97.3 (94.2-99.0)	60.0 (32.3-83.7)	93.5 (89.5-96.3)	14 (5.40-36)	0.64 (0.47-0.88)
**PF4** (134 pg/ml)	83.3 (62.6-95.3)	93.7 (89.7-96.5)	58.8 (40.7-75.4)	98.1 (95.2-99.5)	13 (7.72-23)	0.18 (0.07-0.44)
**sVCAM-1** (46000 pg/ml)	79.2 (57.9-92.9)	81.1 (75.3-86.0)	31.2 (19.9-44.3)	97.3 (93.8-99.1)	4.2 (2.9-5.9)	0.26 (0.12-0.56)
**sVCAM-1** (103700 pg/ml)	66.7 (44.7-84.4)	95.5 (91.9-97.8)	61.5 (40.6-79.8)	96.4 (93.0-98.4)	15 (7.6-29)	0.35 (0.20-0.62)

PPV, Positive Predictive Value, NPV, Negative Predictive Value, +LR, Positive likelihood ratio, -LR, Negative likelihood ratio.

### Identifying the Optimal Biomarker Combination to Accurately Detect ALN

Given that none of the 4 urinary biomarkers by themselves had excellent operating characteristics, we analyzed whether different combinations and/or numbers of elevated biomarkers could more accurately identify ALN patients (**Table 4**). The operating characteristics for any combination of 2 elevated biomarkers were good, with a sensitivity and specificity above 90%, high NPV (99%) and excellent -LR (0.09). These results were similar to those for Adiponectin alone. Even though this combination failed to detect 2 ALN patients, the onset of the LN flare was 12 months prior to the sample collection for both patients and both had a CR at 24 months. This finding suggests that these criteria could rule out ALN cases in a more reliable manner than Adiponectin alone. Nevertheless, the PPV of this combination remained low (50%), with 22 false positives.

**Table 4:** Operating characteristics for different combinations and number of elevated urinary biomarkers to accurately detect ALN patients.

Biomarkers	Operating characteristics calculated using sVCAM cut-off of 46000					
	Sensitivity (95% CI)	Specificity (95% CI)	PPV (95% CI)	NPV (95% CI)	+LR (95% CI)	-LR (95% CI)
2 Elevated biomarkers^*^	91.7 (73.0-99.0)	90.1 (85.4-93.7)	50.0 (34.6-65.4)	99.0 (96.5-99.9)	9.25 (6.11-14)	0.09 (0.02-0.35)
**Different combinations**
Adiponectin-MCP-1	33.3 (15.6-55.3)	100 (98.4-100)	100 (63.1-100)	93.3 (89.3-96.1)	NA	0.67 (0.50-0.88)
Adiponectin-PF4	75.0 (53.3-90.2)	97.3 (94.2-99.0)	75.0 (53.3-90.2)	97.3 (94.2-99.0)	28 (12-63)	0.26 (0.13-0.51)
MCP-1-PF4	37.5 (18.8-59.4)	100 (98.4-100)	100 (66.4-100)	93.7 (89.8-96.4)	NA	0.62 (0.46-0.84)
sVCAM-1- Adiponectin	70.8 (48.9-87.4)	93.2 (89.1-96.2)	53.1 (34.7-70.9)	96.7 (93.4-98.7)	10 (6.04-4.18)	0.31 (0.17-0.58)
sVCAM-1- MCP-1	33.3 (15.6-55.3)	99.1 (96.8-99.9)	80.0 (44.4-97.5)	93.2 (89.2-96.1)	37 (8.33-164)	0.67 (0.51-0.89)
sVCAM-PF4	70.8 48.9-87.4)	96.9 (93.6-98.7)	70.8 (48.9-97.4)	96.9 (93.6-98.7)	22 (10-49)	0.30 (0.16-0.56)
**3 Elevated biomarkers^#^**	70.8 (48.9-87.4)	98.2 (95.5-99.5)	81.0 (58.1-94.6)	96.9 (93.7-98.7)	39 (14-107)	0.30 (0.16-0.55)
**Different combinations**
Adiponectin-MCP-1-sVCAM-1	29.2 (12.6-51.1)	100 (98.4-100)	100 (59.0-100)	92.9 (88.9-95.8)	NA	0.71 (0.54-0.91)
Adiponectin-MCP-1-PF4	33.3 (15.6-55.3)	100 (98.4-100)	100 (63.1-100)	93.3 (89.3-96.1)	NA	0.67 (0.50-0.88)
MCP-1-sVCAM-1-PF4	33.3 (15.6-55.3)	100 (98.4-100)	100 (63.1-100)	93.3 (89.3-96.1)	NA	0.67 (0.50-0.88)
	**Operating characteristics calculated using sVCAM cut-off of 103700**					
**Biomarkers**	**Sensitivity** **(95% CI)**	**Specificity** **(95% CI)**	**PPV** **(95% CI)**	**NPV** **(95% CI)**	**+LR** **(95% CI)**	**-LR** **(95% CI)**
2 Elevated biomarkers^*^	87.5 (67.6-97.3)	94.6 (90.8-97.2)	63.6 (45.1-79.6)	98.6 (95.9-99.7)	16.2 (9.15-29)	0.13 (0.05-0.38)
**Different combinations**
sVCAM-1-Adiponectin	58.3 (36.6-44.9)	97.8 (94.8-99.3)	73.7 (48.8-90.9)	95.6 (92.1-97.9)	26 (10-66)	0.43 (0.27-0.68)
sVCAM-1-MCP-1	33.3 (15.6-55.3)	99.1 (96.8-99.9)	80.0 (44.4-97.5)	93.2 (89.2-96.1)	37 (8.33-164)	0.67 (0.51-0.89)
sVCAM-1-PF4	62.5 (40.6-81.2)	98.7 (96.1-99.7)	89.3 (59.6-96.4)	96.1 (92.6-98.2)	46 (14-148)	0.38 (0.23-0.64)
**3 Elevated biomarkers^#^**	62.5 (40.6-81.2)	99.1 (96.8-99.9)	88.2 (63.6-98.5)	96.1 (92.7-98.2)	69 (17-285)	0.38 (0.23-0.63)
**Different combinations**
Adiponectin-MCP-1-sVCAM-1	29.2 (12.6-51.1)	100 (98.4-100)	100 (59.1-100)	92.9 (88.9-95.8)	NA	0.71 (0.54-0.91)
MCP-1-sVCAM-1-PF4	33.3 (15.6-55.3)	100 (98.4-100)	100 (63.1-100)	93.3 (89.3-96.1)	NA	0.67 (0.50-0.88)

*Any 2 of the 4 biomarkers elevated, ^#^Any 3 of the 4 biomarkers elevated. PPV, Positive Predictive Value; NPV, Negative Predictive Value; +LR, Positive likelihood ratio; -LR, Negative likelihood ratio; NA, Not Applicable due to perfect specificity.

When analyzing the different combinations of 2 elevated biomarkers. The MCP-1-Adiponectin and MCP-1-PF4 combinations had specificities and PPVs of 100%, the combination of Adiponectin-PF4 and MCP-1-sVCAM-1 had a lower PPVs, but still excellent +LRs. sVCAM-1-Adiponectin and sVCAM-1-PF4 combinations had good +LRs but had the lowest PPVs.

Overall, the sensitivities for the individual combinations of 2 biomarkers were not excellent, all below 80% and some as low as 30-40%, suggesting that all 4 biomarkers should be tested in other to improve sensitivity (**Table 4**).

Given that the combinations of 2 elevated biomarkers including sVCAM-1 had lower PPV, we assessed whether increasing the cut-off for sVCAM-1 from 46000 to 103700 improved the operating characteristics. By doing this, the PPV and +LR of the combinations of sVCAM-1-Adiponectin and sVCAM-1-PF4 substantially improved as seen in **Table 4**. The number of false positives for the presence of any combination of 2 elevated biomarkers decreased from 22 (sVCAM-1 cut-off of 46000) to 12 (sVCAM-1 cut-off of 103700). Of the remaining 12 false positives, 9 had RLN and 3 were NLN patients. From the RLN group, 4 had their last LN flare ≤ 2 years before the study (1 of which developed a subsequent flare 2 years later), 3 had chronic proteinuria (all with CKD, 1 of which also had type 2 diabetes mellitus), and 1 had an active urinary tract infection which required antibiotic therapy.

The presence of 3 biomarkers above the established cut-off, irrespective of the combination and the cut-off of sVCAM-1, had excellent specificity, PPV and +LR (**Table 4**). There were only 3 false positives, all with RLN, 2 of whom had their last LN within 2 years of their urine sampling, 1 of whom developed a subsequent flare in the following 2 years. The remaining false positive had an active urinary tract infection. However, this combination had a low NPV (81%) and a -LR above the optimal set point of 0.1 (0.3). Indeed, 6 of 24 ALN patients did not meet this criterion.

### A Two-Step Approach Provides the Best Accuracy for Detecting ALN Patients

**Figure 3:**
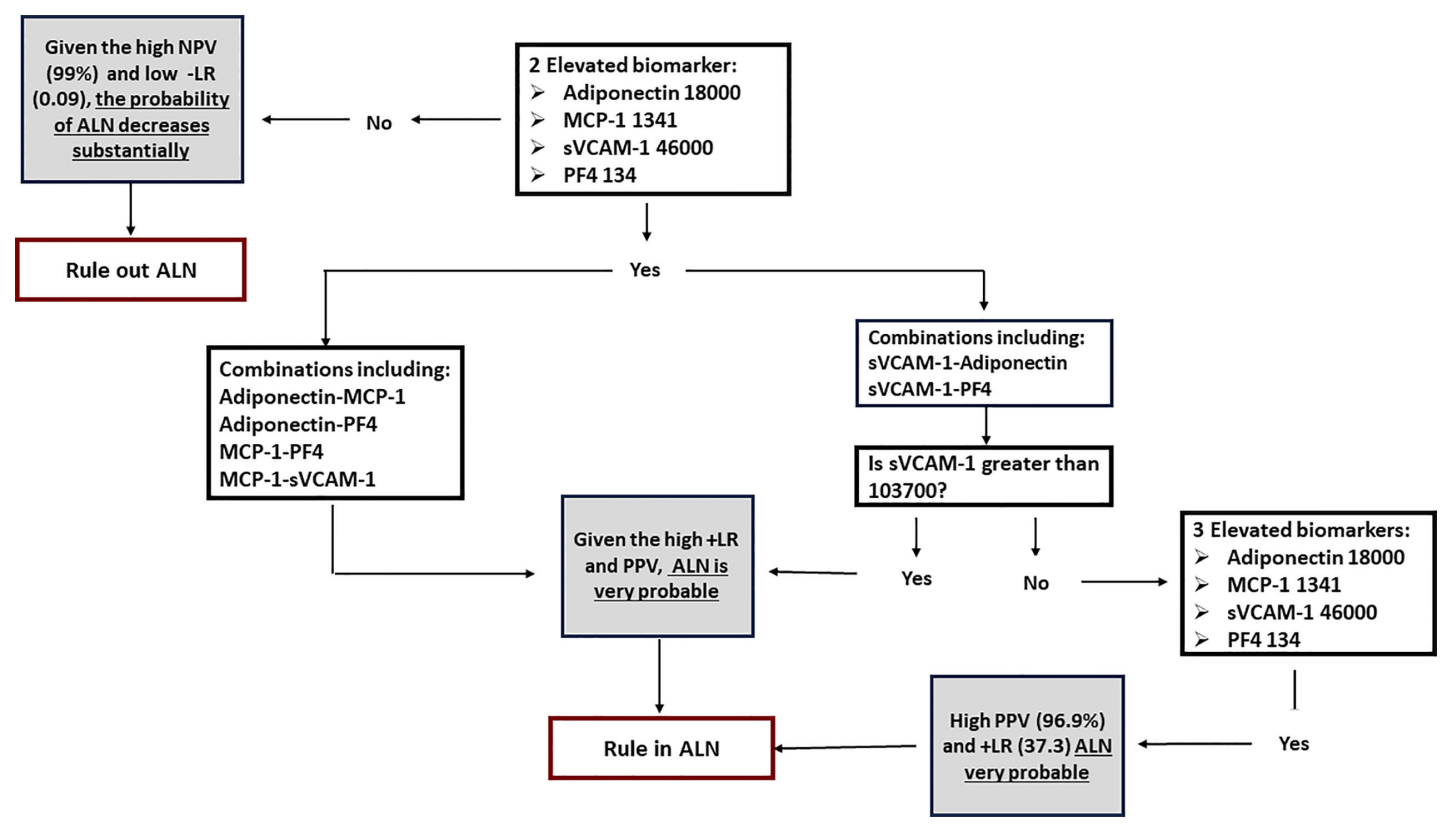
Proposed 2 step approach for the detection of ALN patients.

Based on our results we propose a two-step approach to improve the accuracy of ALN identification (**Figure 3**). In the first step, we propose the following “rule out ALN” criteria. If there are < 2 elevated biomarkers using the lower cut-off for sVCAM-1 (46,000), given the low -LR (0.09) and high NPV (99%), the probability of ALN reduces substantially. For the “rule in ALN” criteria we suggest the following approach. If 2 biomarkers are elevated including the following combinations Adiponectin-MCP-1, Adiponectin-PF4, MCP-1-PF4 and sVCAM-1-MCP-1, given the PPV and +LR, the diagnosis of LN is very probable. On the other hand, if the combination of 2 elevated biomarkers includes sVCAM-1-Adiponectin and sVCAM-1-PF4, then in order to improve accuracy we suggest increasing the cut-off of sVCAM-1 from 46000 to 103700. If there are 3 or more elevated biomarkers, irrespective of the combination and sVCAM-1 cut-off, taking into consideration the high PPV (96.9%) and +LR (37.3), LN is very likely. Urinary tract infections should be ruled out, as they may cause false positive results.

### Our Proposed “Rule Out ALN Criteria” at 12 Months Following ALN Flare Predicts Response to Treatment at 24 Months

In the first stage of the study, we determined that the percentage decrease of Adiponectin, MCP-1, sVCAM-1, PF4, vWF and IL-15 after 12 ± 3 months of treatment predicted response to therapy at 24 months. In order to evaluate if our rule out criteria (presence of < 2 elevated urinary biomarkers, with sVCAM-1 cut-off of 46,000) could also serve as a predictor of response to treatment we analyzed a subpopulation of the cross-sectional cohort who´s urine sample was collected at 12 ± 3 months after their LN flare. Of the twenty-two patients in the, analysis, 12 had < 2 elevated biomarkers, with 11 achieving a CR at 24 months. In contrast only 4 out of 10 patients with ≥ 2 elevated biomarkers achieved a CR (p= 0.02, Fisher´s exact test). The operating characteristics for this subpopulation analysis were as follows: sensitivity (73.3 [95%CI 44.9-92.2]), specificity (85.7 [95%CI 42.1-99.6]), PPV (91.7 [95%CI 61.5-99.8]) and NPV (60.0 [95%CI 26.2-87.8]).

### Our “Rule in ALN” Criteria Operate Better for Proliferative ALN

To corroborate the sensitivity of our “rule in ALN” criteria established in the cross-sectional cohort and determine if they operate similarly for proliferative and non-proliferative LN classes, we measured urinary Adiponectin, MCP-1, sVCAM and PF4 in a biopsy-proven ALN cohort. A total of 53 patients were included, of whom 35 had proliferative LN and 18 non-proliferative class (4 with class V and chronic proliferative LN and 14 with pure II or V LN classes). **Supplementary Table 2** shows their demographic and clinical characteristics at the time of the urine collection. As seen in **Table 5**, the sensitivity of our “rule in ALN” criteria was similar for the group of proliferative ALN, 91.4% for the presence of 2 or more elevated biomarkers (higher sVCAM cut-off of 103,700) and 77.1% for the presence of 3 or more elevated biomarkers (sVCAM cut-off of 46,000). However, the sensitivities were much lower for the non-proliferative classes, suggesting that our “rule in ALN” criteria work better for proliferative LN.

**Table 5:** Sensitivity of our “rule in ALN” criteria in a biopsy-proven ALN cohort.

	Sensitivity (95%CI) using sVCAM-1 cut-off of 46000 pg/ml		
	Whole cohort (N = 53)	Proliferative LN (N = 35)	Non-Proliferative LN (N = 18)
**2 Elevated biomarkers**	81.1 (68.0-90.6)	91.4 (76.9-98.2)	61.1 (35.8 – 82.7)
**3 Elevated biomarkers**	66.0 (51.3-78.8)	77.1 (59.9-89.6)	55.6 (30.8-78.5)
	**Sensitivity (95%CI) using sVCAM-1 cut-off of 103,700 pg/ml**		
**2 Elevated biomarkers**	79.3 (65.9-89.2)	91.4 (76.9-98.2)	55.6 (30.8-78.5)
**3 Elevated biomarkers**	56.6 (42.3-70.2)	62.9 (44.9 – 78.5)	55.6 (30.8-78.5)

## Discussion

Given the invasive nature of the KB, the current gold standard for LN diagnosis, and the known drawbacks of proteinuria, the most commonly used parameter for LN surveillance, there is a tremendous need for biomarkers that accurately identify active LN cases. In this study we identified 4 urinary biomarkers that not only discriminate between ALN and non-ALN patients, but also have the capacity to reflect clinical improvement, and we have proposed “rule out” and “rule in” criteria that accurately detect proliferative ALN patients. Importantly, this can be accomplished relatively inexpensively using conventional ELISAs at an approximate cost of only 6.90 CAD per sample to perform all 4 urinary biomarkers. All 4 biomarkers, Adiponectin, MCP-1, sVCAM-1 and PF4, have been previously proposed by our group and others as potential biomarkers for ALN (15–24). In addition, prior studies have shown that all 4 analytes correlate with the activity index in the kidney biopsy (16, 29, 30), suggesting that they play a role in LN pathogenesis.

Higher levels of serum Adiponectin have been found in ALN (15, 31). While the pathogenic role of Adiponectin in LN is still unclear, several studies support an anti-inflammatory (32) and even reno-protective (33) action. However, it has been shown that the low molecular weight isoform of Adiponectin, has pro-inflammatory properties (34–37). This finding suggests that under inflammatory conditions the predominant isoform could shift converting Adiponectin’s action from anti-inflammatory to proinflammatory.

MCP-1 is induced by type I interferons and multiple pro-inflammatory cytokines (38). MCP-1 has potent chemiotactic activity, especially for macrophages and neutrophils (39–41), and thus may act to promote leucocyte recruitment to the kidney. Consistent with this possibility, it was shown to increase prior to proteinuria in LN flares (42) and higher levels are associated with worse clinical outcomes (20, 42–45).

Serum and urinary levels of sVCAM-1, a surrogate marker for endothelial expression of VCAM and endothelial activation (46, 47), have been shown to be elevated in SLE and to correlate with overall disease activity and the presence and severity of LN (29, 30, 48–51). sVCAM-1 can also serve as a chemotactic stimulus for monocytes (52) and T lymphocytes (53). Hence, sVCAM-1 may be responsible for the recruitment, adhesion and transmigration of multiple phagocyte cells to the kidney (54).

PF4 has been implicated as a possible urinary biomarker for LN in several studies (16, 21). PF4 is mainly released by activated platelets and is an inflammatory response mediator with potent chemotactic, especially for monocytes and neutrophils (55–57)

In the first stage of our study, 4 urinary biomarkers were chosen from 15 studied analytes based on their property to decrease with therapy and accurately discriminate between complete responders and non-responders to therapy. These results were further validated when we analyzed the subpopulation of 22 patients from the cross-sectional cohort who were diagnosed with ALN 12 months prior to the urine collection, where most of the patients who achieved CR at 24 months had < 2 elevated biomarkers (our “rule out” ALN criteria). Our results are in accordance with the study by Brunner et al, where they showed that decreased levels of several urinary biomarkers, including Adiponectin and MCP-1, could predict treatment responses (58). However, in contrast to our study they found that several of these could predict responses, as early as 3 months following initiation of therapy. There were 2 key differences between that study and ours. Firstly, Brunner et al. studied LN in children and young adults, and secondly, many of their patients were treated with cyclophosphamide, whereas the majority of our patients were treated with mycophenolate. It is likely that these treatment differences explain the differences in time course for the response of the biomarkers to therapy, as shown in a sub-analysis of their data in which they contrasted patients treated with cyclophosphamide and mycophenolate, where the mycophenolate data showed similar delayed responses to those seen with our patients.

In the second stage of the study, based on the operating characteristics from our established cut-offs and the number of elevated biomarkers needed to accurately detect ALN cases, we propose a 2-step approach for the classification of ALN. Our “rule out” ALN criteria had an excellent NPV and -LR. The operating characteristics for the “rule in ALN” criteria were also very good. Even though the CI for the PPV´s were relatively wide, which could be a consequence of the small sample size of ALN patients, the lower limit of the CI of the +LR´s were all close to or above 10, indicating that the presence of LN is very likely.

The “rule out ALN” criteria included 2 ALN patients, both of whom started their flare 12 months prior to the sample collection and achieved CR at year 2. As was shown in **Figure 1**, most of the patients that demonstrated a CR at year 2, had normalized their urinary biomarker levels at year 1, thus, it is not surprising that these 2 ALN patients were not detected by our test. Furthermore, it has been demonstrated that up to 60% of patients who are still clinically active (proteinuria) have no histologic activity in repeat kidney biopsies, hence it’s possible that these 2 patients that were included in our ALN group based on our ALN criteria were instead inactive, although without a kidney biopsy we can only speculate.

Most of the false positives for the “rule in ALN” criteria were patients with RLN who had a relatively recent LN flare (2 years prior), subsequent flares, or chronic proteinuria that was interpreted by their physician as secondary to damage. Prior studies have demonstrated that up to 30% of the patients who achieve clinical remission after induction therapy will continue with histologic activity on repeat kidney biopsies (14) and that active histologic findings may continue for up to 2 years or longer after a LN flare, even in the absence of significant proteinuria (59). Hence, it is possible that these ‘false positive’ patients had ongoing kidney inflammation. It is notable that our cohort included 9 patients with chronic proteinuria and 2 more with CKD (with no proteinuria), and only 3 (27%) of these met the “rule in criteria”, suggesting that in general, elevated levels of our urinary biomarkers do not simply reflect kidney damage and may add relevant information to proteinuria for the detection of active LN. Given the lack of a KB at the time of the urine collection, we cannot definitively determine if the elevated biomarkers in these cases reflect kidney inflammation or are true false positives.

Recent data indicate that a KB performed two years after the LN flare can provide important clinical information, with residual kidney inflammatory activity forecasting subsequent LN flares (59). Our findings indicate that a subset of patients with RLN have elevated levels of inflammatory factors and that some of these will develop a subsequent flare. It will be important to correlate our urinary biomarkers with repeat KBs to determine if these biomarkers could serve as potential surrogates for ongoing kidney activity, which could eliminate the need for a repeat KB.

In addition to RLN, 3 patients with NLN met the “rule in ALN” criteria. Of these, 2 had high levels of sVCAM-1, and had high titers of antiphospholipid antibodies, one with antiphospholipid syndrome (APS) and recurrent thrombosis, and the other with relapsing episodes of vasculitis. Increased expression of soluble adhesion molecules, including sVCAM-1, has been demonstrated in patients with APS (60). Furthermore, sVCAM-1 has been suggested as a prognostic marker of clinical complications in APS including abortions, repeated thrombosis and kidney involvement (60, 61), which could account for the elevated levels of this urinary biomarker in these patients. Another important aspect to consider when using the “rule in criteria” is the presence of urinary infections that could certainly cause false positives and should be ruled out.

As the cohorts for stage 1 and 2 of the study included predominately proliferative ALN patients, we further validated the sensitivity of our “rule in ALN” criteria in a biopsy-proven ALN cohort, of whom around 30% had non-proliferative classes. The sensitivity of our criteria was similar in the proliferative group but substantially lower for the non-proliferative classes. These results are not surprising considering that the pathogenic role of all 4 biomarkers is more in keeping with proliferative LN. In addition, we and other groups have shown that all 4 analytes correlate with the activity index in the kidney biopsy (16, 29, 30). A strength of our study is that we validated the proposed biomarkers in three independent cohorts and by 2 different methods of detection, demonstrating the reproducibility of their discriminatory and predictive abilities. In addition, the unbiased patient recruitment of the cross-sectional cohort reflected a real-life scenario, with LN patients at different stages of their flare, which allowed us to create cut-offs that may be more sensitive to detect ongoing lower grade activity.

This study has several limitations. The sample size of ALN patients in our second cross-sectional cohort was small and the majority of the ALN patients in both cohorts had proliferative LN. A further limitation is the lack of KBs at the time of the urine collection for many of the LN patients, making it difficult to conclude that false negative patients lacked renal inflammation and conversely that false positive patients were truly false positives. To account for these limitations, we further validated the sensitivity of our “rule in ALN” criteria in a biopsy-proven ALN cohort that included non-proliferative ALN patients. We recognize that our cut-offs and criteria to detect ALN need to be externally validated in an independent cohort.

In summary our results provide further evidence to support the role of Adiponectin, MCP-1, sVCAM-1 and PF4 in the detection of proliferative ALN cases. In addition, we show the clinical utility of measuring multiple rather than a single biomarker. Finally, we propose a novel “rule in” and “rule out” approach for the detection of proliferative ALN with excellent operating characteristics which may provide additional information beyond proteinuria for the detection of proliferative ALN patients and monitoring the response to treatment.

## Data Availability Statement

The original contributions presented in the study are included in the article/**Supplementary Material**. Further inquiries can be directed to the corresponding author.

## Ethics Statement 

The studies involving human participants were reviewed and approved by Research Ethics Board of the University Health Network. The patients/participants provided their written informed consent to participate in this study.

## Author Contributions

PF, ZT, KG, LW-G, and JW were responsible for study conception and design. LW-G, KG, MK, DB, DG, MU, ZT, PF, and JW were responsible for the acquisition of data. EA, LW-G, and JW performed the data analysis and interpretation. All authors were involved in drafting the article or revising it critically for important intellectual content, and all authors approved the final version to be published.

## Funding

This study was funded by a New Emerging Team grant from the Canadian Institutes of Health Research (CIHR - QNT #78341) to PF and JW, a CIHR Proof-of Principle Grant (PPP-144247), and the Bruce Beauchamp Fund. PF is supported by a tenured Professorship of Medicine and a Canada Research Chair on systemic autoimmune rheumatic diseases at Université Laval. LW-G is recipient of the Lupus Foundation of America, Gary S. Gilkeson MD career award, ZT is supported by an Arthritis Society Young Investigator Award and a Canadian Rheumatology Association (CIORA) - Arthritis Society Clinician Investigator Award. JW is supported by The Arthritis Centre of Excellence of the University of Toronto, a Pfizer Chair Career Award, and the Schroeder Arthritis Institute. Support for this study came also from the Lupus Program, Centre for Prognosis Studies in the Rheumatic Diseases. The funders were not involved in the study design, collection, analysis, interpretation of data, the writing of this article or the decision to submit it for publication.

## Conflict of Interest

The authors declare that the research was conducted in the absence of any commercial or financial relationships that could be construed as a potential conflict of interest.

## Publisher’s Note

All claims expressed in this article are solely those of the authors and do not necessarily represent those of their affiliated organizations, or those of the publisher, the editors and the reviewers. Any product that may be evaluated in this article, or claim that may be made by its manufacturer, is not guaranteed or endorsed by the publisher.
