# Supplementary material for: Identification and Validation of a Urinary Biomarker Panel to Accurately Diagnose and Predict Response to Therapy in Lupus Nephritis
Source: Front Immunol. 2022 May 30;13:889931. doi: 10.3389/fimmu.2022.889931 (PMC9196040; doi:10.3389/fimmu.2022.889931)

Supplementary Material

**Supplementary Figure 1.** Receiver Operating Characteristic curves for our 4 candidate urinary biomarkers.


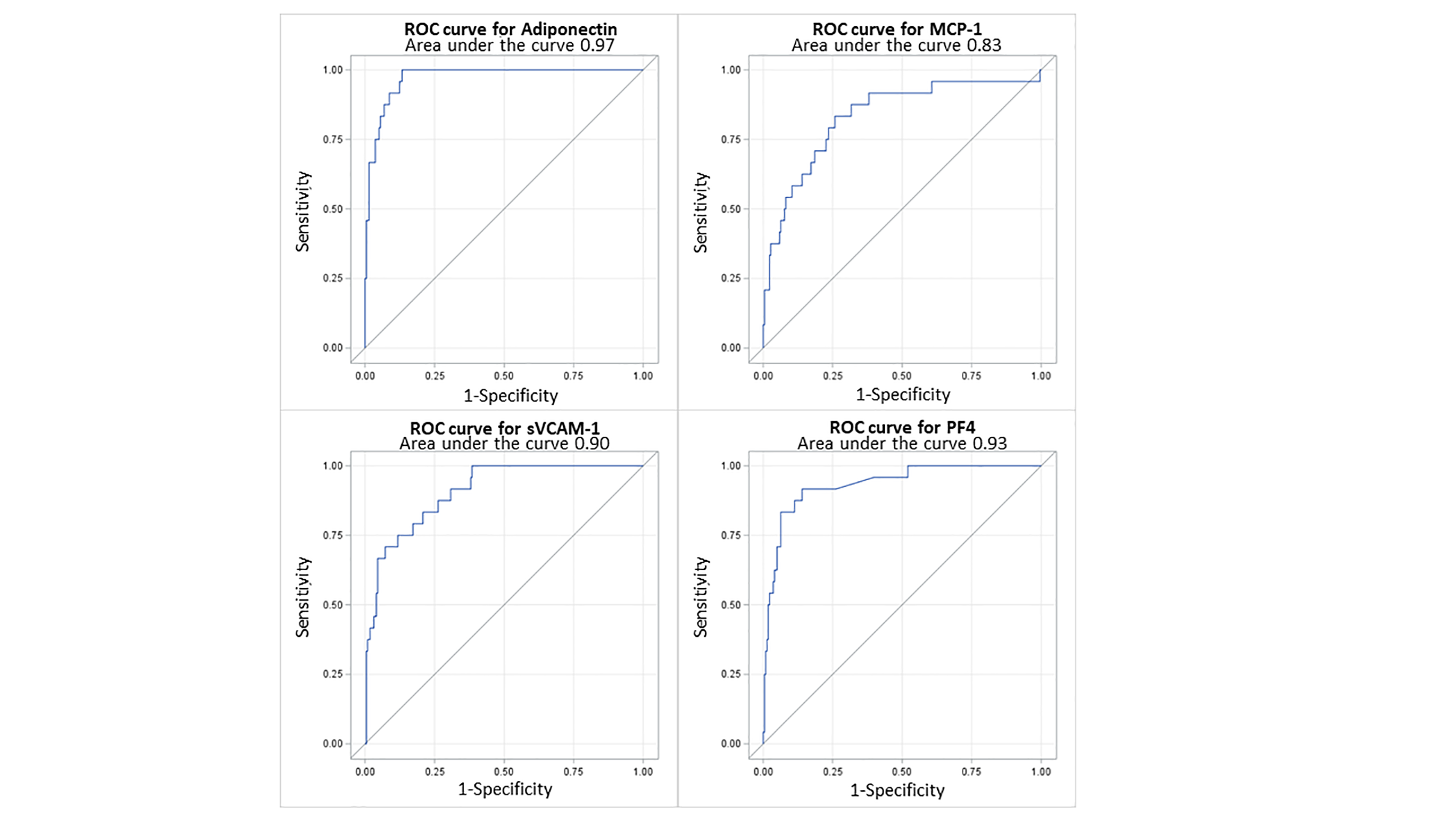

Supplement: Supplementary file 1 [file DataSheet_1.docx]
