# Supplementary material for: Identification and Validation of a Urinary Biomarker Panel to Accurately Diagnose and Predict Response to Therapy in Lupus Nephritis
Source: Front Immunol. 2022 May 30;13:889931. doi: 10.3389/fimmu.2022.889931 (PMC9196040; doi:10.3389/fimmu.2022.889931)
Supplement: Supplementary file 2 [file Table_1.docx]

Supplementary Material

| **Supplementary Table 1. Distribution of Biomarkers depending on their activity status: ALN, RLN and NLN. Data expressed as Median (IQR).** | | | | |
| --- | --- | --- | --- | --- |
|  | **ALN (N=24)** | **RLN (N=79)** | **NLN (N=144)** | **P-value** |
| **Adiponectin**  **pg/ml** | 46888.3 (24387.8, 78227.1) | 5183.0 (2285.6, 10900.7) | 3566.4 (1329.7, 8377.3) | <.0001 |
| **PF4**  **pg/ml** | 321.7 (138.0, 1547.2) | 35.3 (26.1, 44.2) | 30.3 (26.6, 39.4) | <.0001 |
| **MCP-1**  **pg/ml** | 771.7 (291.4, 1780.7) | 125.6 (50.5, 300.3) | 100.0 (47.8, 236.0) | <.0001 |
| **sVCAM-1**  **pg/ml** | 125754.7 (62505.9, 268615.7) | 18383.7 (7046.1, 46158.2) | 9530.2 (2636.6, 25781.2) | <.0001 |
