# Supplementary material for: Identification and Validation of a Urinary Biomarker Panel to Accurately Diagnose and Predict Response to Therapy in Lupus Nephritis
Source: Front Immunol. 2022 May 30;13:889931. doi: 10.3389/fimmu.2022.889931 (PMC9196040; doi:10.3389/fimmu.2022.889931)
Supplement: Supplementary file 3 [file Table_2.docx]

| **Supplementary Table 2. Baseline demographic and clinical characteristics of the biopsy-proven ALN cohort^†^. N=53** | |
| --- | --- |
| **Ethnicity, n (%)** |  |
| Caucasian | 23 (43.4) |
| Afro-Caribbean | 13 (24,5) |
| Asian | 9 (16.9) |
| Other | 8 (15.1) |
| **Female, n (%)** | 46 (86.7) |
| **Age (years), Median (IQR)** | 26.70 (22.3-42.0) |
| **Duration SLE (years), Median (IQR)** | 3.00 (0.1-9.1) |
| **Time from LN flare (months)*, Median (IQR)** | 1.0 (0-2.0) |
| **SLEDAI, total score, Median (IQR)** | 16.0 (9.0-22.0) |
| **SLEDAI, renal, Median (IQR)** | 8.0 (4.0-12.0) |
| **Anti-dsDNA Ab (IU/ml), Median (IQR)** | 77.0 (9.0-100.0) |
| **C3, g/rL, Median (IQR)** | 0.66 (0.52-0.82) |
| **C4, gr/L, Median (IQR)** | 0.07 (0.07-0.21) |
| **Serum Albumin (gr/L), Median (IQR)** | 29.0 (22.0-34.0) |
| **Serum Creatinine (umol/L), Median (IQR)** | 131.0 (59.5-151.0) |
| **24-hour Protein excretion (gr), Median (IQR)** | 3.02 (1.3-3.6) |
| **Kidney biopsy Class, n (%)** | 53 (100) |
| I | 1 (1.8) |
| II | 3 (5.6) |
| III | 4 (7.5) |
| IV | 18 (33.9) |
| V | 10 (18.8) |
| III+V | 4 (7.5) |
| IV + V | 13 (24,5) |
| VI | 0 (0) |
| **Activity Index, Median (IQR)** | 10.0 (7.5-13.0) |
| **Chronicity Index, Median (IQR)** | 3.0 (2.0-5.0) |
| **Prednisone, n (%)** | 52 (98.1) |
| **Prednisone dose (mg), Median (IQR)** | 40.0 (30.0-50.0) |
| **Antimalarial, n (%)** | 49 (92.4) |
| **Immunossuppressive, n (%)** | 53 (100) |
| **Azathioprine, n (%)** | 9 (16.9) |
| **Mychophenolate, n (%)** | 34 (64.1) |
| **Cyclophosphamide, n (%)** | 7 (13.2) |
| **Calcineurin Inhibitors, n (%)** | 3 (5.6) |
| **^†^**Baseline clinical characteristics are at the time of the urine sample collection, *Time from LN flare to urine sample collection (months). | |
